# Classroom-Based Micro-Sessions of Functional High-Intensity Circuit Training Enhances Functional Strength but Not Cardiorespiratory Fitness in School Children—A Feasibility Study

**DOI:** 10.3389/fpubh.2019.00291

**Published:** 2019-11-14

**Authors:** Florian A. Engel, Matthias Oliver Wagner, Franziska Schelhorn, Felix Deubert, Sascha Leutzsch, Alexander Stolz, Billy Sperlich

**Affiliations:** ^1^Department Movement and Training Science, Institute of Sport and Sport Science, Heidelberg University, Heidelberg, Germany; ^2^Department of Sport Science, Bundeswehr University Munich, Neubiberg, Germany; ^3^Integrative and Experimental Training Science, Institute for Sport Sciences, University of Würzburg, Würzburg, Germany

**Keywords:** aerobic fitness, exercise intervention, functional training, interval training, physical activity promotion

## Abstract

The present study assessed the short-term effect of 6 min classroom-based micro-sessions of multi-joint functional high-intensity circuit training (Functional_HIIT_) performed by students during regular classes on parameters related to functional strength and cardiorespiratory fitness. In this randomized controlled 4-week study, 17 students (11 male; 6 female; age: 11.6 ± 0.2 years) performed 6 min of Functional_HIIT_ (targeting >17 on the Borg scale) 4 days per week during regular school classes and 18 students (11 male; 7 female; age: 11.7 ± 0.3 years) served as control group (CG) without any additional in-class physical activity. The Functional_HIIT_ group completed 86% of all planned sessions (mean duration: 6.0 ± 1.5 min) with a mean RPE of 17.3 ± 2.1. Body height, mass and BMI did not differ between the groups at baseline or between pre- and post-testing (*p* > 0.05; eta^2^ ≤ 0.218). The performances in lateral jumping (*p* < 0.000; part eta^2^ = 0.382; Δ% 4.6 ± 8.6), sit-ups (*p* < 0.000; part eta^2^ = 0.485; Δ% 3.1 ± 8.6) and 20-m sprints (*p* < 0.000; part eta^2^ = 0.691; Δ% 15.8 ± 5.4) improved in both groups with greater increase following Functional_HIIT_. No baseline differences and no interaction effects occurred in performance of 6 min run, flexibility, push-ups, balance, and long jump. Classroom-based Functional_HIIT_ sessions, performed 4 days per week during 4 weeks did not improve variables related to aerobic endurance performance but enhanced certain parameters of functional strength in schoolchildren. As time is limited in the educational system of schools, Functional_HIIT_ during regular school classes could offer a new perspective for increasing functional strength in schoolchildren.

## Introduction

Youth levels of physical inactivity is increasing in Europe (1) and worldwide (2) with 70–90% of European adolescents failing recommended levels of physical activity (PA) (2) and with high sedentary behavior (1). A low level of PA may result in poor cardiorespiratory fitness (CRF) while low PA and CRF are both considered as risk factors for developing cardiovascular diseases (CVD) (3–5) and obesity (3–6). While most recommendations for regular PA of children and adolescents refer to moderate-to-vigorous activity of 60 min daily (7), repeated bouts of high-intensity exercise [i.e., high-intensity interval training (HIIT)] demonstrated time-efficient (8–10) and positive effects for improving CRF in (non-)obese children and adolescents with most interventions ranging from 7 to 10 weeks with 2–3 sessions per week (9–13).

Since children spend 40% of their waking hours in the school (14) it seems the school setting is ideal to implement PA promotion. In this context HIIT was recently applied within physical education (PE) classes of children (15, 16) and adolescent students (17, 18) to enhance CRF [determined by a shuttle run (17, 18) and 6 min walking test (16)]. Additionally, HIIT not only improved CRF but also functional strength when applied for relative short periods of 7–10 weeks with 1–3 sessions/week (19, 20). PE pursues additional objectives other than exclusively improving CRF, functional strength or motor performance (i.e., sport specific skill acquisition, motor learning, and development of personal-, social- and methodological competences). Thus, PE as school subject *per se* and due to time constraints seems restricted to improve CRF, functional strength and motor performance sustainably. Because of its proven time-efficiency and effectiveness in improving the key variables CRF and short functional strength bouts of HIIT could be applied during or in-between regular (non PE) classes within the academic classroom sparing enough time for other academic aims.

Usually HIIT protocols are running- (20–23) or cycling-based (22, 24, 25), requiring either space or equipment. As a special form of HIIT circuit-like, multiple-joint, high-intensity exercises (Functional_HIIT_), performed daily during 4 weeks (26), respectively three sessions per week during 9 weeks (27), have been applied successfully to improve CRF and functional strength, body composition as well as certain aspects of quality of life in adults. Functional_HIIT_ does not require a lot of space or equipment and is therefore applicable in any academic classroom. A recent large scale study demonstrated that, independent of the time and patterns of sedentary behavior, moderate-to-vigorous physical activity is associated with health-related physical fitness in 13-year old children (28). Therefore, we assume that micro-sessions of Functional_HIIT_ may contribute to improve health-related physical fitness and ultimately reduce the risk for cardiovascular diseases in children even though Functional_HIIT_ (lasting e.g., 5–8 min) does not fulfill the current recommendations for PA. Although time-efficient 6 min Functional_HIIT_ proved to increase muscular strength and perceived quality of life in untrained adults (26) it is astonishing that this type of exercise has not been applied in an academic classroom during school hours to evaluate whether Functional_HIIT_ improves CRF and functional strength in school children. Therefore, the aim of this study was to evaluate the effects of a 6 min circuit-like, multiple-joint high-intensity interval training performed within regular school classes in the academic classroom of 11–12 year old school children vs. a group performing no additional physical activity in the classroom.

The potential benefits of implementing Functional_HIIT_ interventions in the academic classroom include (i) bypassing “lack of time” as one reason for not exercising (29) and (ii) the high degree of adherence to the PA interventions at school.

We hypothesized that the Functional_HIIT_ group would significantly improve cardiorespiratory fitness and functional strength from baseline (T_0_) to after the four-week intervention (T_1_) in comparison to the control group.

## Materials and Methods

### Participants

In this single-center, two-arm randomized, controlled study, *n* = 35 secondary school children (24 male; 11 female; age: 11.7 ± 0.3 years) of the south of Germany participated (Table 1).

**Table 1:** Anthropometric parameters (means ± SD) for the Functional_HIIT_ (*n* = 17) and control group (*n* = 18) before (T_0_) and after (T_1_) the 4-week intervention.

**Parameter**	**Group**	**T_**0**_**	**T_**1**_**	***p***	**η^2^**	**F**	***p***	**η^2^**	**F**
				**(T)**			**(T** **×** **G)**		
Body height [m]	Functional_HIIT_	1.57 ± 0.07	1.58 ± 0.06	0.424	0.021	0.801	0.104	0.011	0.415
	Control	1.58 ± 0.07	1.58 ± 0.07						
Body mass [kg]	Functional_HIIT_	40.7 ± 7.0	41.3 ± 6.7	0.725	0.015	0.327	0.676	0.004	0.182
	Control	46.0 ± 10.0	46.2 ± 9.9						
BMI [kg·m^−2^]	Functional_HIIT_	16.4 ± 2.1	16.5 ± 2.0	0.119	0.218	0.436	0.184	0.028	0.143
	Control	18.2 ± 2.7	18.5 ± 4.1						
Age [years]	Functional_HIIT_	11.6 ± 0.2	11.6 ± 0.2	–	–	–	–	–	–
	Control	11.7 ± 0.3	11.7 ± 0.3						

*p, probability; η^2^, effect size partial eta-square; T, global effect of time; T × G, global effect of Time × Group; F, degrees of freedom*.

During the 4 week intervention, *n* = 17 (11 male; 6 female; age: 11.6 ± 0.2 years) students performed 4 days per week one supervised 6 min micro-session of Functional_HIIT_ (Table 2) during their regular school class and 18 students (11 male; 7 female; age: 11.7 ± 0.3 years) served as control group without additional in-class exercise. All participants were free to withdraw from the study at any time without further consequences. The inclusion criteria were: (i) an age of 10–12 years; (ii) no frequent participation in endurance or strength exercise programs for at least 6 months prior to the study; (iii) no daily intake of medication; and (iv) for inclusion in the analysis, completion of at least 80% of all possible training sessions.

**Table 2:** Details of the 4-week Functional_HIIT_ training intervention.

**Week**	**Session**	**Functional_**HIIT**_**
1	1	– 2 series of – 30-s squats + 15-s recovery – 30-s lunges + 15-s recovery – 3 series of – 50-s skippings + 10-s recovery
	2	– 2 series of – 20-s push-ups + 10-s recovery – 20-s crunches + 10-s recovery – 20-s dips + 10-s recovery – 3 series of – 50-s skippings + 10-s recovery
	3	– 45-s jumping jacks + 15-s recovery – 45-s plank + 15-s recovery – 2 series of – 20-s burpees + 10-s recovery – 3 series of – 50-s skippings + 10-s recovery
2	1	– 2 series of – 30-s squats + 15-s recovery – 30-s lunges + 15-s recovery –3 series of – 50-s skippings + 10-s recovery
	2	– 2 series of – 20-s push-ups + 10-s recovery – 20-s crunches + 10-s recovery – 20-s dips + 10-s recovery – 3 series of – 50-s skippings + 10-s recovery
	3	– 45-s jumping jacks + 15-s recovery – 45-s plank + 15-s recovery – 2 series of – 20-s burpees + 10-s recovery – 3 series of – 50-s skippings + 10-s recovery
	4	– 2 series of – 30-s squats + 15-s recovery – 30-s lunges + 15-s recovery – 3 series of – 50-s skippings + 10-s recovery
3	1	– 2 series of – 20-s push-ups + 10-s recovery – 20-s crunches + 10-s recovery – 20-s dips + 10-s recovery – 3 series of – 50-s skippings + 10-s recovery
	2	– 45-s jumping jacks + 15-s recovery – 45-s plank + 15-s recovery – 2 series of – 20-s burpees + 10-s recovery – 3 series of – 50-s skippings + 10-s recovery
	3	– 2 series of – 30-s squats + 15-s recovery – 30-s lunges + 15-s recovery – 3 series of – 50-s skippings + 10-s recovery
	4	– 2 series of – 20-s push-ups + 10-s recovery – 20-s crunches + 10-s recovery – 20-s dips + 10-s recovery – 3 series of – 50-s skippings + 10-s recovery
4	1	– 45-s jumping jacks + 15-s recovery – 45-s plank + 15-s recovery – 2 series of – 20-s burpees + 10-s recovery – 3 series of – 50-s skippings + 10-s recovery
	2	– 2 series of – 30-s squats + 15-s recovery – 30-s lunges + 15-s recovery – 3 series of – 50-s skippings + 10-s recovery
	3	– 2 series of – 20-s push-ups + 10-s recovery – 20-s crunches + 10-s recovery – 20-s dips + 10-s recovery – 3 series of – 50-s skippings + 10-s recovery
	4	– 2 series of – 30-s jumping jacks + 15-s recovery – 30-s squats + 15-s recovery – 3 series of – 50-s skippings + 10-s recovery

All procedures were conducted in accordance with the Declaration of Helsinki (30). The experimental protocol was pre-approved by the ethical review board of the Sport Science Institute of the University of Würzburg. All students and their legal guardians as well as their teachers were informed in detail about the design of the study, including the potential risks and benefits, before providing their written informed consent to participate.

#### Overall Study Design

**Figure 1:**
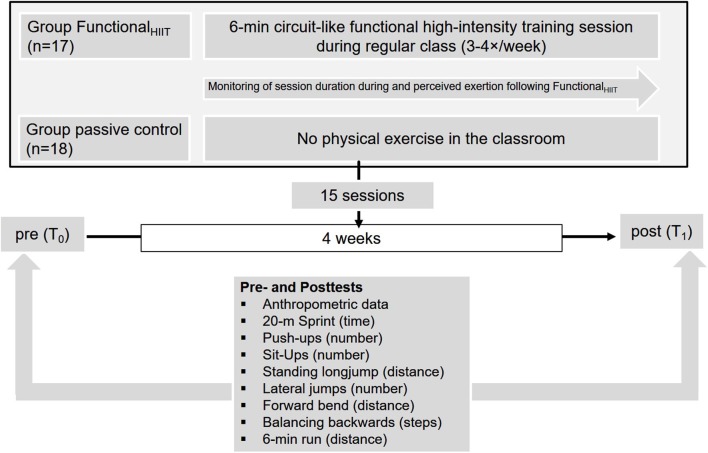
The study design, including testing prior to (T_0_) and following the intervention period (T_1_).

At baseline (T_0_) and after the four-week intervention (T_1_), all students underwent assessment of body height and mass. Functional strength and CRF were assessed using the “German Motor Ability Test” (31). The post-intervention assessment (T_1_) took place 72 h after the final scheduled Functional_HIIT_ session (Figure 1). All assessments during T_0_ and T_1_ were performed in the gym and track and field court of the school, supervised by the principal investigators, teacher and trained undergraduate students. The German Motor Ability Test shows a very good independence from the examiner, with a mean correlation coefficient for all eight tests of *r* = 0.95 (range: 0.86–0.99). Test-Retest reliability shows a mean correlation coefficient for all eight tests of *r* = 0.82 (31).

The school was chosen since the school offered a sufficient number of students and one researcher had contact to the school before the start of the study.

#### Functional_HIIT_

In week 1 participants performed three training sessions, during week 2–4 participants performed four Functional_HIIT_ sessions per week (Table 2). During the 4-week intervention period participants performed different 6 min micro-sessions of Functional_HIIT_ (Table 2) in the academic classroom during school hours. Functional_HIIT_ involved 6 min circuit-like, multiple-joint exercises with high-intensity interval training protocols and all exercises were performed with the children's own bodyweight and rather high velocities and repetitions (Table 2). During each Functional_HIIT_ session the teacher demonstrated Functional_HIIT_ movements (for details, see Table 2 and www.sportsandscience.de, 2017a; b; c; d). The children were instructed to follow these movements with “all-out” effort targeting >17 on the 6−20 Borg scale (32). Children aged 11–14 demonstrated being able to regulate their exercise intensity independently to correspond to intensities of 17 (very hard) using the 6–20 Borg scale (33). The teacher gave feedback to students considering the correct execution of Functional_HIIT_ movements and provided standardized verbal encouragement.

#### Anthropometric Data and Body Composition

Height was measured with a portable stadiometer (seca 213, seca, Hamburg, Germany). Body mass was assessed with an electronic scale (seca 799, seca, Hamburg, Germany). Height and body mass were measured while participants wore sports clothes and no shoes. The body-mass-index was calculated as body mass/(body height)^−2^.

#### German Motor Ability Test

The German Motor Ability Test was performed as described in detail elsewhere (31) and has been applied frequently to assess CRF and functional strength (34–36). As mentioned above, the German Motor Ability Test shows very good test quality criteria for all test items (31). This type of testing is frequently applied in German PE so that all children of the present study were accustomed to all procedures. Briefly, the German Motor Ability Test comprises of eight different subtests:

- *20-m sprint*: All participants sprinted a 20-m distance as fast as possible. Time was measured with single beam timing lights (Brower Timing Systems, Draper, USA) and used for statistical analysis.
- *Standing long jump*: All children jumped once from standing position with both legs as far as possible. The covered distance employed for statistical analysis.
- *Forward bend*: All children stood on a bench and bended their trunk forward toward their feet with straight elbows and knees. The deepest position of the fingertips was obtained for statistical analysis.
- *Lateral jumping*: Participants completed within 15 s as many lateral jumps from one side of a square (50 × 100 cm) to another. The number of correctly performed jumps was employed for statistical analysis.
- *Balancing backwards*: All children balanced backwards on bars of three different widths (consecutively: 6.0, 4.5, and 3.0 cm). The number of steps before touching the ground (maximum *n* = 8) per bar were obtained for statistical analysis.
- *Sit-Ups*: All performed as many sit-ups as possible within 40-s. The number of correct repetitions within 40-s were counted for statistical analysis.
- *Push-ups*: Participants performed as many push-ups as possible within 40-s. The number of correctly performed repetitions within 40-s were counted for statistical analysis.
- *6 min run*: All participants ran or walked constantly around a volleyball court (9 × 18 m) for 6 min. The covered distance of each participant was obtained for statistical analysis.

#### Ratings of Perceived Exertion

Participants were familiarized with the Borg 6–20 RPE scale before the start of the intervention (32). Immediately after each Functional_HIIT_ session, all students rated their perceived exertion during exercise on the Borg 6–20 scale (32). Children aged ~11 years were found capable of expressing exercise intensities on the RPE scale as precise as adults (37). For analysis, we averaged the data from all participants over all Functional_HIIT_ sessions. Before the start of the study we assess the heart rate response of ten children during Functional_HIIT_ to ensure the heart rate would increase to >85% of heart rate max. Since we could not apply heart rate monitoring during regular school classes (due to time constraints) we employed RPE as surrogate for the training intensity.

### Statistical Analyses

All data of T_0_ and T_1_ were confirmed to be normally distributed by the Kolmogorov-Smirnov test and homogeneity of variance (Levene Test) were tested prior to further statistical analysis, so that no transformation was required. A two way repeated-measures ANOVA [time-point (T_0_, T_1_) × group (Functional_HIIT_, CG)] was performed for each outcome variable, with an alpha of *p* < 0.05 being considered statistically significant and indicated by asterisk. The values obtained were evaluated by calculating the effect size partial eta-square (η^2^) (38) in order to evaluate practical relevance, with η^2^ ≥ 0.01 indicated small, ≥ 0.06 medium and ≥ 0.14 large effects (39). Additionally, data of T_0_ was checked for significant differences between the two groups, applying student's *t*-test for unpaired samples. G^*^power software (40) was used to calculate the required sample size and based on the study of Sperlich et al. (26) it was determined that 20 participants per group would be required to provide sufficient power to detect statistically significant effects. The means and standard deviations (*SD*) for all data sets were calculated and all statistical tests were carried out in SPSS 22.0 software (SPSS Inc., Chicago, IL, USA).

## Results

### Training Adherence, Training Duration, Perceived Exertion

All students of the Functional_HIIT_ group achieved the required minimum of 80% of scheduled training with 86% sessions completed during the 4-week intervention. No participant dropped out during the 4-weeks intervention. Two participants were excluded from analyses due to absence during the post-testing. The mean duration of Functional_HIIT_ sessions was 6.0 ± 1.5 min, the corresponding mean value for RPE was 17.3 ± 2.1.

### Pre-post Testing

All parameters obtained at T_0_ and T_1_ and statistical analyses are presented in Tables 1, 3. All parameters measured at T_0_ did not differ between the two groups (lowest *p* ≥ 0.113; lowest η^2^ ≥ 0.003). All anthropometric data did not differ between T_0_ to T_1_ and groups (lowest *p* ≥ 0.104; lowest η^2^ ≥ 0.004).

**Table 3:** Parameters of functional strength and endurance (means ± SD) for the Functional_HIIT_ and Controle group before (T_0_) and after (T_1_) the 4 week intervention.

**Parameter**	**Group**	**T_**0**_**	**T_**1**_**	**Δ**	**Δ%**	***p***	**η^2^**	**F**	***p***	**η^2^**	**F**
						**(T)**			**(T** **×** **G)**		
Push-ups [n]	Functional_HIIT_	19.4 ± 2.6	20.6 ± 3.4	1.2 ± 2.8	9.9 ± 15.6	0.054	0.108	3.997	0.453	0.017	0.576
	Control	19.0 ± 3.8	19.6 ± 3.4	0.6 ± 2.6	5.3 ± 19.4						
Sit-ups [n]	Functional_HIIT_	26.5 ± 2.7	27.4 ± 3.7	0.9 ± 2.4	3.1 ± 8.6	0.001^*^	0.283	13.005	0.000^*^	0.485	31.133
	Control	28.4 ± 4.7	24.4 ± 4.3	−4.0 ± 2.6	−14.0 ± 8.3						
Standing long jump [cm]	Functional_HIIT_	160 ± 1.4	165 ± 1.3	5.0 ± 1	3.4 ± 4.4	0.000^*^	0.318	15.4	0.485	0.015	0.499
	Control	157 ± 1.8	160 ± 1.8	4.0 ± 1	2.3 ± 4.2						
Lateral jumps [n]	Functional_HIIT_	41.5 ± 4.3	43.5 ± 6.0	1.9 ± 3.6	4.6 ± 8.6	0.141	0.065	2.277	0.000^*^	0.382	20.400
	Control	42.6 ± 5.8	38.6 ± 3.1	−4.0 ± 4.1	−8.3 ± 10.0						
20-m sprint [s]	Functional_HIIT_	3.93 ± 0.28	3.31 ± 0.25	−0.63 ± 0.23	−15.8 ± 5.4	0.000^*^	0.754	101,383	0.000^*^	0.691	73.694
	Control	4.01 ± 0.25	3.96 ± 0.18	−0.05 ± 0.17	−1.1 ± 4.3						
6 min run [m]	Functional_HIIT_	1124 ± 123	1143 ± 90	19 ± 51	2.1 ± 4.7	0.255	0.039	1.341	0.615	0.008	0.258
	Control	1055 ± 139	1063 ± 106	8 ± 84	1.5 ± 9.1						
Flexibilty [cm]^†^	Functional_HIIT_	−4.6 ± 8.8	−4.3 ± 8.2	0.3 ± 3.1	−3.3 ± 91.5	0.167	0.057	1.994	0.055	0.107	3.974
	Control	−3.5 ± 8.4	−5.1 ± 8.0	−1.6 ± 2.9	−33.2 ± 93.0						
Balance [steps]	Functional_HIIT_	2.5 ± 0.6	2.4 ± 0.7	−0.1 ± 0.7	4.9 ± 55.5	0.715	0.004	0.135	0.992	0.000	0.000
	Control	2.2 ± 0.8	2.2 ± 1.0	−0.1 ± 1.1	7.8 ± 69.0						

P, probability; η^2^, effect size partial eta-square; T, global effect of time; T × G, global effect of Time × Group; F, degrees of freedom;

* T_1_ differs significantly from T_0_;

† *negative values indicate a better flexibilty*.

The performances in lateral jumping [effect of time × group: *F*_(1, 33)_ = 20.40; *p* < 0.000; part eta^2^ = 0.382], sit-ups [effect of time × group: *F*_(1, 33)_ = 31.13; *p* < 0.000; part eta^2^ = 0.485) and 20-m sprint [effect of time × group: *F*_(1, 33)_ = 73.69; *p* < 0.000; part eta^2^ = 0.691) increased in both groups with greater improvements in Functional_HIIT_ vs. control group. All interactions revealed large effect sizes (eta^2^ = 0.382 −0.691). The total difference in performance at T_1_ between Functional_HIIT_ and control group (difference in performance = performance Functional_HIIT_ – performance control group) were: lateral jumps: 4.9 [n]; sit-ups: 3.0 [n]; 20-m sprint: 0.65 [s].

The performance in 6 min run [effect of time × group: F_(1, 33)_ = 0.258; *p* < 0.615; part eta^2^ = 0.008], flexibility [effect of time × group: *F*_(1, 33)_ = 3.974; *p* < 0.055; part eta^2^ = 0.107], push-ups [effect of time × group: *F*_(1, 33)_ = 0.576; *p* < 0.453; part eta^2^ = 0.017), balance [effect of time × group: *F*_(1, 33)_ = 0.000; *p* < 0.992; part eta^2^ = 0.000) as well as standing long jump [effect of time × group: *F*_(1, 33)_ = 0.499; *p* < 0.485; part eta^2^ = 0.015) revealed no significant changes over time and between groups (see Table 3). The absolute difference in performance between Functional_HIIT_ and control group was: 6 min run: 80.0 [m]; flexibility: 0.8 [cm]; push-ups: 1.0 [n]; balance: 0.2 [steps]; standing long jump: 5.0 [cm].

## Discussion

The main findings of the present study concerning the responses of variables related to functional strength and CRF of untrained 11-year-old students to either 4 weeks of classroom-based Functional_HIIT_ or passive control condition with no exercise were as follows:

(i) performance in lateral jumping, sit-ups and 20-m sprint were greater after 4 weeks Functional_HIIT_ compared to the control group;

(ii) in both groups no changes occurred after 4 weeks in 6 min run, flexibility, push-ups, balance as well as standing long jump;

(iii) all anthropometric parameters remained unchanged between groups and over time.

In contrast to CRF, the parameters related to functional strength (lateral jumping, sit-ups, 20-m sprint) exhibited significant interaction effects in favor of Functional_HIIT_ group, demonstrating the effectiveness of Functional_HIIT_ for the improvement of functional strength. Since muscular fitness is inversely associated with cardiovascular disease risk factors and improvements in muscular fitness and sprinting speed seem to demonstrate a positive effect on skeletal health in children and adolescents (3), the present Functional_HIIT_ intervention may reveal beneficial health effects. Overall, following Functional_HIIT_ the adaptations in functional strength (lateral jumping, sit-ups, 20-m sprint) were higher than those of CRF. These finding are in line with other studies performing similar HIIT sessions with children at school (41, 42) as well as with adults in other settings (26, 27). These findings underline that functional strength performance is responsive for adaptions following a short exercise intervention such as the present Functional_HIIT_. The pronounced increase in functional strength following Functional_HIIT_ may be due to adaptations in neuro-muscular structure and function, e.g., release of inhibitory mechanisms, as well as improvements in intra- and intermuscular coordination (synchronization, recruitment, and the rate coding of muscle fibers) with improved neural adaptations (43, 44). It seems that the short duration and the four training sessions per week of Functional_HIIT_ are a potent stimulus to promote equivalent increases in neuro-muscular function when compared to other traditional strength training programs (45).

The present results are consistent with other findings demonstrating the positive impact of power and strength training on functional strength as well as on jumping and sprinting performance in children and adolescents in different settings including physical education (44, 46, 47).

Surprisingly, the control group also demonstrated improvements from T_0_ to T_1_ for some test items (e.g., push-ups: 5.3%; standing long jump: 2.3%; flexibility: 33.2%; balance: 7.8%). Since the control group performed no additional physical exercise during the classes, it is possible that improvements occured due to adaptations to regular physical education classes, extracurricular sports engagement or due to developmental effects. However, since all anthropometric parameters remained unchanged between groups and over time it is unlikely that developmental effects caused the improvements. Since the German Motor Ability Test demonstrates a high mean correlation coefficient for all eight test items of *r* = 0.82 (31), we suggest that it is more likely that improvements of the control group arose from regular PE classes or extracurricular sports engagement. However, since the study did not control for PA level we cannot identify the cause for performance improvements with certainty.

From a practical perspective and in contrast to traditional apparatus-based strength training the present Functional_HIIT_ did not require any barbells, devices or machinery. All exercises were performed with the children's own bodyweight and rather high velocities and repetitions. The changes in functional strength after 4 weeks represent a good cost-benefit ratio since no expensive equipment was necessary—a prerequisite for school-based sports activities. Since the space requirement for Functional_HIIT_ is very low (all exercises comprised mainly vertical movements in an area of about 1 m^2^ per student) the chosen Functional_HIIT_ in connection with the low space requirements favor this type of exercise as classroom based PA.

Despite the current HIIT-related research in children and adolescents (9, 10, 12, 13, 48) only few classroom- or school- based HIIT micro-sessions have been examined with scientific rigor (41, 42). Therefore, it is difficult to determine which components of HIIT protocols [e.g., interval intensity and duration, rest intensity and duration, exercise modality, number of repetitions, number of series, between-series recovery duration and intensity (49)] maybe superior for improving dimensions of functional strength and CRF of young students.

In contrast to other studies dealing with HIIT and young and adolescent students in a school-based environment (16, 17, 20, 21, 41, 48, 50, 51) the present study demonstrated no significant effects on CRF (6 min run performance) by Functional_HIIT_ and several reasons may explain this result. The short session durations of Functional_HIIT_ (6.0 ± 1.5 min) combined with the short intervention period (4 weeks) as well as the exercise mode of Functional_HIIT_ (i.e., the movements were more related to functional strength) may not have evoke a sufficient stimulus to improve CRF in the participating children. Other school-related HIIT studies improved peak oxygen uptake and employed a longer intervention periods (≥6 weeks) and/or session durations (10, 15, 19, 34, 40, 41) ranging from approximately 14 min (17), 40 min (10, 15, 40) up to 60 min (20). In a recent review Braaksma et al. (11) recommended for improving CRF with PA interventions in children from 6 to 12 years a duration of at least 6 weeks and a frequency of 3–4 times per week. Most probably, our intervention did not improve CRF because each Functional_HIIT_ sessions and overall intervention period were too short. While an extension of the present intervention period (4 weeks) seems feasible, we consider an extension of session duration (6.0 ± 1.5 min) of classroom-based interventions as unfavorable.

In the light of the present results, we cannot recommend 6 min Functional_HIIT_ in order to improve CRF in students. Running-based protocols with high or maximum intensities similar to recent studies (16, 20, 48, 50, 51) seem more appropriate exercise than 6 min Functional_HIIT_ to improve CRF in students. Most studies showing improved CRF in young individuals incorporate running (21–23) or cycling (22, 24, 25) based HIIT protocols. It seems these protocols achieve higher stimuli, which trigger cardio-respiratory adaptations more effectively than Functional_HIIT_.

The Functional_HIIT_ sessions of the present study took place during regular classes and therefore replaced 6 min of sedentary behavior with 6 min of intensive physical exercise. Consequently, daily Functional_HIIT_ sessions during regular classes may have the potential to reduce the amount of sedentary time and may contribute to increase the general level of physical activity in children.

The adherence to the current intervention of Functional_HIIT_ was quite high (>86% of all planned sessions), which is not surprising since the exercise program took place during regular classes. Nevertheless, the classroom teacher had no concerns that Functional_HIIT_ session would be disruptive in any form for achieving the curricular aims of the class and therefore approved the intervention beforehand. This high degree of adherence in students and teachers may reflect the fact that the intervention was relatively short and detailed, as well as the fact that the individual sessions did not take much time. In this regards the present study is in line with a very similar study of Ekström et al. (41) who reported that teachers confirmed a good integration of a daily 4 min exercise intervention in the classroom.

Considering the intensity of Functional_HIIT_, students were instructed to perform the Functional_HIIT_ between 17 and 20 on the Borg scale, whereas 20 represents “all-out” intensity. Since the mean intensity of Functional_HIIT_ sessions was 17 ± 2, the RPE scale did reflected the lower range and not the higher range of the intended high-intensity effort.

## Limitations

A greater number of participants would have given more statistical power to the data interpretation with less risk of calculating type 2 errors. At the same time, the degree to which these results can be generalized is limited due to the small number of participants. The study shows an imbalanced ratio of male and female participants, which represents a limitation since effects of PA interventions may depend on sex in children (52, 53). Furthermore, the small sample size does not warrant an analysis for the potential influence of sex or age on the results. However, we included two school classes since (i) the present study represents a first pilot study to test feasibility and adaptions to Functional_HIIT_ and (ii) a lower sample size allows better controlling of Functional_HIIT_ and increase availability, motivation, and compliance of students during participation.

The inclusion of a physical activity enjoyment scale could have given information on the enjoyment of Functional_HIIT_ and may had identified if enjoyment of Functional_HIIT_ had mediated the relationship between the exercise program and the outcome.

In order to describe the study participants precisely, some studies applying HIIT with children and/or adolescents (54, 55) incorporate assessment of sexual maturation of participants [e.g., Tanner stages (56)]. Due to personal and organizational restrictions, this was not possible in the present study.

Initially we aimed to record heart rate during the Functional_HIIT_ sessions and we aimed to record Functional_HIIT_ sessions as well as leisure time with accelerometers. However, we refrained of employing this methodology because of the frequent (un)dressing with heart rate straps and the accelerometers.

## Conclusion

Four weeks with 3–4 session per week of 6 min Functional_HIIT_ performed during regular classes do not enhance CRF (assessed by 6 min test) but improve certain dimensions of functional strength (lateral jumping, sit-ups, 20-m sprint) in 11-year-old students. The Functional_HIIT_ intervention improves 20-m sprint, lateral jumps and sit-ups to a higher extent compared to a passive in-class control condition. Consequently, the present intervention of Functional_HIIT_ represents a feasible, sufficient, and effective exercise intervention, which enhances health relevant muscular fitness among young students and could be added as a complement to regular physical education and curricular learning activities during regular classes. As time is limited in the educational system of schools, Functional_HIIT_ during regular school classes may offer a novel and feasible intervention for increasing functional strength in young students. Since Functional_HIIT_ did not improve CRF in children, we recommend performing this type of exercise with greater intensity, longer session duration as well as interventional period.

## Data Availability Statement

The datasets used and/or analyzed during the current study are available from the corresponding author on reasonable request.

## Ethics Statement

All procedures were conducted in accordance with the Declaration of Helsinki (30). The experimental protocol was pre-approved by the ethical review board of the Sport Science Institute of the University of Würzburg.

## Author Contributions

All authors listed have made a substantial, direct and intellectual contribution to the work, and approved it for publication.

### Conflict of Interest

The authors declare that the research was conducted in the absence of any commercial or financial relationships that could be construed as a potential conflict of interest.
